# Long-term outcomes of Kono-S anastomosis for ileocecal resections in Crohn’s disease: a comparative analysis

**DOI:** 10.1007/s00384-026-05098-7

**Published:** 2026-02-02

**Authors:** Maximilian Vojta, Maike Hermann, Peter Kienle, Christoph Reißfelder, Christel Weiß, Julia Hardt, Steffen Seyfried

**Affiliations:** 1 Department of Surgery, Medical Faculty Mannheim, University Medical Center Mannheim, Heidelberg University, Theodor-Kutzer-Ufer 1-3, 68167 Mannheim, Germany; 2 Department of Surgery, Brüderklinikum Julia Lanz, Mannheim, Germany; 3 Department of Biometry and Statistics, Medical Faculty Mannheim, University Medical Center Mannheim, Heidelberg University, Mannheim, Germany

**Keywords:** Crohn’s disease, Kono-S anastomosis, Ileocecal resection, Long-term outcomes, Quality of life, Surgical timing

## Abstract

**Objective:**

This long-term follow-up study evaluates clinical and functional outcomes after ileocecal resection with either Kono-S or conventional anastomosis techniques in patients with Crohn’s disease. The goal was to determine whether the Kono-S approach confers a long-term advantage in preventing disease recurrence and improving quality of life.

**Summary of background data:**

While the Kono-S anastomosis has shown promise in reducing recurrence rates in Crohn’s disease following surgery, most existing evidence stems from short- to medium-term follow-up. High-quality long-term data remain scarce, particularly in real-world clinical settings. This study aims to fill that gap.

**Methods:**

A retrospective-prospective cohort analysis was performed including patients who underwent ileocecal resection for Crohn’s disease between 2015 and 2017 at a single academic center. Patients were grouped according to anastomosis technique (Kono-S vs. conventional). Long-term follow-up data were obtained via chart review, imaging studies, and patient-reported questionnaires, including the Gastrointestinal Quality of Life Index (GIQLI). Primary outcomes included recurrence rates, postoperative complications, and quality of life.

**Results:**

Seventy patients were included in the final analysis (Kono-S: *n* = 31; conventional: *n* = 39). The median follow-up duration was 8.1 years (interquartile range = 6.9–8.8 years). No significant differences were observed between groups regarding endoscopic inflammation (Kono-S = 19.4%, conventional = 25.6%, *p* = 0.39), restenosis (Kono-S = 9.7%, conventional = 2.6%, *p* = 0.34), or GIQLI scores (Kono-S: median 116 vs. 110, *p* = 0.08). Rehospitalization rates were numerically higher in the Kono-S group (16.1% vs. 2.6%, *p* = 1.0), but not statistically significant. Importantly, approximately 40% of all patients retrospectively stated they would have preferred earlier surgical intervention, independent of the anastomotic technique.

**Conclusion:**

After more than 7 years of follow-up, the Kono-S anastomosis demonstrates comparable long-term outcomes to conventional techniques in terms of recurrence, complications, and quality of life. Patient reflections suggest a potential benefit of earlier surgical intervention, highlighting the need for more proactive surgical referral in gastroenterological practice.

## Introduction

Despite substantial progress in medical management strategies for Crohn’s disease (CD)—including the widespread use of immunosuppressants and biologic agents—surgical intervention remains an unavoidable reality for a considerable portion of patients. Epidemiological data indicate that as many as 80% of individuals diagnosed with CD will require at least one intestinal resection during the course of their disease [1, 2]. Notably, recurrence of the disease, particularly at the site of the anastomosis, remains a persistent challenge. This underscores the critical importance of evaluating how various surgical techniques may influence postoperative recurrence and long-term clinical outcomes [3, 4].

In this context, a novel approach to anastomosis was introduced by Kono et al. in 2011, known as the Kono-S anastomosis. This technique was specifically developed to address the high-risk mesenteric border—recognized as a common site for disease recurrence. The Kono-S approach involves constructing an antimesenteric, functional, end-to-end, hand-sewn anastomosis, which theoretically minimizes exposure to mesenteric inflammation. Preliminary studies have reported promising outcomes, suggesting that this configuration significantly reduces postoperative recurrence rates when compared with traditional methods [5].

However, these favorable initial findings have been primarily based on short- to medium-term follow-up data. Long-term outcomes remain insufficiently explored, particularly in real-world clinical settings. Robust evidence from extended follow-up periods is essential to validate the initial benefits observed and to support broader clinical adoption of this technique.

The objective of the present study is to perform a comprehensive long-term evaluation of the Kono-S anastomosis in patients undergoing ileocecal resection for Crohn’s disease. Specifically, we aim to assess and compare its efficacy relative to conventional anastomosis techniques by analyzing recurrence rates, postoperative complications, and overall patient-reported quality of life.

## Materials and methods

This study was designed as a single-center ambispective (retrospective–prospective) observational cohort study conducted at the University Medical Center Mannheim, Germany. The study was approved by the local ethics committee (Ethics Approval ID: 2017-575N-MA) and conducted in accordance with the Declaration of Helsinki. Written informed consent was obtained from all participants included in the long-term follow-up.

### Participants

All consecutive adult patients who underwent ileocecal resection for histologically confirmed Crohn’s disease between January 2015 and December 2017 were eligible for inclusion.

**Fig. 1:**
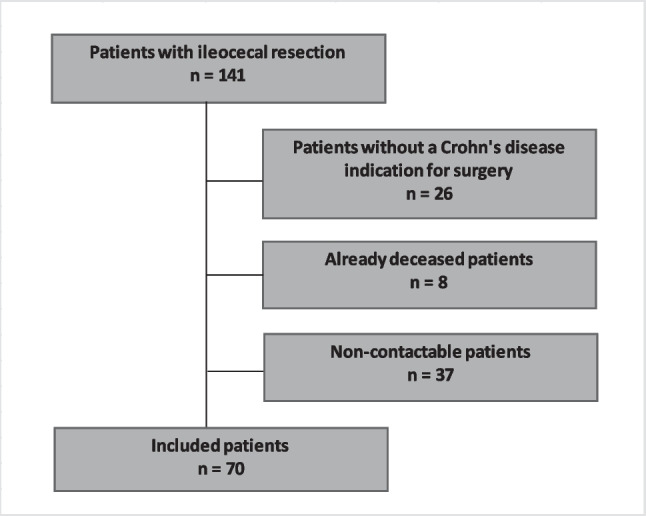
Study design

A total of 141 patients underwent ileocecal resection during the study period. After exclusion of patients without confirmed Crohn’s disease, deceased patients, and patients lost to follow-up, 70 patients were available for final analysis (Fig. 1).

Long-term follow-up data were collected between December 2024 and February 2025, resulting in a follow-up duration ranging from 7 to more than 10 years after index surgery.

Operative technique:

Patients were categorized according to the anastomotic technique used at the index operation:

1. Kono-S anastomosis, defined as an antimesenteric functional end-to-end hand-sewn anastomosis.
2. Conventional anastomosis, defined as an anisoperistaltic extracorporeal side-to-side stapled functional anastomosis using a linear stapler, occasionally reinforced with a single-layer extramucosal hand-sewn closure.

The choice of anastomotic technique was based on surgeon preference and intraoperative considerations and was not randomized.

### Outcomes

The primary outcomes were long-term disease recurrence and functional outcome. Disease recurrence was defined as new endoscopic inflammation in the neoterminal ileum proximal to the anastomosis. Inflammation or complications at other intestinal sites were considered disease progression rather than anastomotic recurrence. Functional outcome was assessed using the Gastrointestinal Quality of Life Index (GIQLI) [6].

Secondary outcomes included anastomotic restenosis, Crohn’s disease–related rehospitalization, need for reoperation or re-resection, postoperative weight change, and short-term perioperative morbidity (operative time, length of hospital stay, and postoperative complications).

### Data sources and measurements

Perioperative data were obtained retrospectively from the institutional surgical database and electronic medical records, including demographic characteristics, operative details, postoperative complications, and length of hospital stay.

Long-term follow-up data were collected prospectively through structured telephone interviews and chart review. Patients were contacted primarily by telephone; if unreachable, up to four contact attempts were made, including contact via relatives or primary care physicians when permitted by data protection regulations. If telephone contact was unsuccessful, a written request was sent by mail.

Clinical follow-up data were derived from available colonoscopy reports and cross-sectional imaging (magnetic resonance imaging or computed tomography) performed as clinically indicated. Body weight change, smoking status, and quality of life were recorded. GIQLI questionnaires were completed either during the telephone interview or returned by mail.

Patients were additionally asked to retrospectively assess the timing of surgery and indicate whether they would have preferred surgery earlier, later, at the same time, or not at all.

All data were pseudonymized and stored on a secure institutional server.

### Bias and missing data

Selection bias is inherent due to the non-randomized allocation of surgical technique based on surgeon preference and patient-specific factors. Follow-up assessments were not standardized and were performed according to clinical indication, which may introduce detection bias. A substantial proportion of patients could not be contacted or had died prior to follow-up, potentially leading to attrition bias. Missing data were not imputed.

### Study size

The study represents an exploratory cohort analysis based on all eligible patients available for long-term follow-up. No formal sample size calculation or power analysis was performed.

### Statistical analysis

Statistical analyses were performed using SAS software (version 9.4, SAS Institute Inc., Cary, NC). Continuous variables were summarized as medians with interquartile ranges or means with ranges, as appropriate. Categorical variables were reported as absolute numbers and percentages.

Comparisons between groups were performed using the chi-square test or Fisher’s exact test for categorical variables. Continuous variables were analyzed using the Wilcoxon rank-sum test for non-normally distributed data or Student’s *t*-test for normally distributed data. A two-sided *p* value < 0.05 was considered statistically significant.

## Results

Out of a total of 141 patients who underwent ileocecal resection during the study period, 70 patients met the inclusion criteria for final analysis after excluding those who had surgery for non-Crohn’s-related indications and those who had deceased (see Fig. 1 for study flowchart). Within this cohort, 31 patients (44%) received a Kono-S anastomosis, while 39 patients (56%) underwent conventional anastomosis techniques.

The detailed baseline characteristics of the long-term follow-up cohort are shown in Table 1. Age, sex distribution, and body mass index (BMI) were comparable between the two groups, as summarized in Table 1. The mean age at the time of surgery was 48.2 years (range = 30–78) in the Kono-S group and 50.7 years (range = 27–75) in the conventional group (*p* = 0.47). BMI values showed no significant difference between groups either, with a mean of 23.4 kg/m^2^ in the Kono-S group and 25.4 kg/m^2^ in the conventional group (*p* = 0.16).

**Table 1:** The baseline characteristics

	Kono-S (*n* = 31)	Conventional (*n* = 39)	*p*-value
*Sex*			0.93
Men	14	18	
Women	17	21	
Age in years, mean (range)	48.2 (30–78)	50.7 (27–75)	0.47
BMI in kg/m^2^, mean (IQR)^1^	23.4 (20.5–24.8)	25.4 (20.3–27.3)	0.16
*Medication*			
Cortisone	4	7	0.97
Mesalazine/Azathioprine	6	5	0.86
Biologicals	8	12	0.43
*Smoking status*			0.59
Non smoker	6	8	
Former smoker	7	13	
Active smoker	17	17	
Not specified	1	1	

^1^Interquartile range (25–75%)

Regarding pharmacological treatment, the distribution of medications prior to surgery—including corticosteroids, mesalazine/azathioprine, and biologics—was generally balanced across the two groups. Similarly, smoking status (non-smoker, former smoker, or current smoker) showed no statistically significant differences (*p* = 0.59), thereby supporting the comparability of the cohorts.

### Short-term outcomes

Operative and early postoperative outcomes are presented in Table 2. The mean operative time was slightly shorter in the Kono-S group at 166.4 min compared to 178.8 min in the conventional group; however, this difference was not statistically significant (*p* = 0.72). The mean duration of postoperative hospital stay was also similar between the groups—9.8 days for the Kono-S group and 11.5 days for the conventional group (*p* = 0.22).

**Table 2:** Operative and early postoperative outcomes

	Kono-S (*n* = 31)	Conventional (*n* = 39)	*p*-value
Operation time in minutes, mean (IQR)	166.4 (140–188.5)	178.8 (140–201)	0.72
Duration of hospital stay in days, mean (IQR)	9.8 (8–9.5)	11.5 (8–12)	0.22
Postoperative complications			
*Overall*	2	1	0.58
Anastomotic insufficiency	1	1	
Hemorrhage	1	0	

Postoperative complications were infrequent in both groups. Two patients in the Kono-S cohort and one patient in the conventional group experienced complications during the immediate postoperative period (*p* = 0.58). One case of anastomotic insufficiency requiring surgical revision occurred in each group. Notably, one patient in the Kono-S group developed a postoperative hemorrhage related to the mesentery, which necessitated additional surgical intervention.

### Long-term outcomes

Median follow-up (time from surgery to long-term follow-up visit) was 2967 days (Q1–Q3 = 2507–3210 days) or 8.1 years (Q1–Q3 = 6.9–8.8 years). Long-term follow-up data, which included endoscopic and radiologic evaluations, revealed no statistically significant differences in disease recurrence or postoperative complications between the two groups, as detailed in Table 3. Colonoscopy follow-up was more frequently performed in the Kono-S group (90.3%) compared to the conventional group (74.3%), while MRI and CT utilization remained similar between cohorts.

**Table 3:** Long-term follow-up data

	Kono-S (*n* = 31)	Conventional (*n* = 39)	
*Follow-up examinations*			
Colonoscopy (%)	28 (90.32%)	29 (74.36%)	
MRI (%)	12 (38.71%)	14 (35.89%)	
CT (%)	1 (3.23%)	4 (10.26%)	
*Pathological findings*			*p*-value
Inflammation	6	10	0.39
Restenosis	3	1	0.34
Re-hospitalization^1^	5	1	1
Re-resections	1	0	0.85
Change in weight in kg, mean (IQR)	2.3 (0–5.8)	6.3 (0–11)	0.27
*Gastrointestinal quality of life*			
GIQLI score, median	116	110	0.08

^1^Hospitalization related to the underlying disease

Endoscopic inflammation at the anastomotic site was observed in 6 patients (19.4%) in the Kono-S group and in 10 patients (25.6%) in the conventional group (*p* = 0.39). Although the Kono-S group showed a slightly lower frequency of inflammatory changes, the difference was not statistically significant. Restenosis was detected in 3 patients in the Kono-S group and 1 patient in the conventional group (*p* = 0.34), again without reaching statistical significance.

Rehospitalizations related to Crohn’s disease occurred in 5 patients in the Kono-S group and in 1 patient from the conventional group. Despite this numerical difference, statistical analysis did not reveal significance (*p* = 1). Regarding postoperative changes in body weight, patients in the Kono-S group had a mean gain of 2.3 kg, while those in the conventional group gained 6.3 kg (*p* = 0.27).

In terms of gastrointestinal quality of life, as assessed by the GIQLI [6] questionnaire, patients in the Kono-S group reported a slightly higher median score (116 vs. 110), suggesting a trend toward better perceived well-being; however, this difference was not statistically significant (*p* = 0.08).

When asked to reflect on the appropriateness of the timing of their surgery, responses from both groups were broadly similar and are presented in Table 4. A majority of patients in both the Kono-S group (51.61%) and the conventional group (41.03%) reported satisfaction with the timing of their operation, stating they would choose the same timing again. A notable proportion in both groups—14 patients (45.16%) in the Kono-S group and 17 patients (42.59%) in the conventional group—indicated that, in retrospect, they would have preferred to undergo surgery earlier. Importantly, no patient in either group expressed a wish that the surgery had been performed later. One patient (3.23%) in the Kono-S group and four patients (10.26%) in the conventional group stated they would have opted against surgery entirely, given their subsequent experience.

**Table 4:** Appropriateness of the timing of surgery

	Kono-S (*n* = 31)	Conventional (*n* = 39)
Same timing of surgery	16 (51.61%)	16 (41.03%)
Earlier surgery	14 (45.16%)	17 (42.59%)
Later surgery	0	0
No operation at all	1 (3.23%)	4 (10.26%)
Not specified	0	2 (5.13%)

## Discussion

In this long-term cohort study with a median follow-up exceeding 8 years, the Kono-S anastomosis demonstrated outcomes comparable to conventional ileocolic anastomotic techniques in patients undergoing ileocecal resection for Crohn’s disease. No statistically significant differences were observed with respect to endoscopic recurrence, anastomotic restenosis, rehospitalization, reoperation, or health-related quality of life.

Although earlier studies reported a significant reduction in endoscopic and clinical recurrence rates with the Kono-S technique, our long-term data did not confirm such a benefit. This finding is consistent with more recent prospective studies that likewise failed to demonstrate a sustained advantage in recurrence prevention [7–9].

Luglio et al. demonstrated a significant advantage of the Kono-S anastomosis with respect to endoscopic and clinical recurrence in the first randomized controlled trial evaluating this technique; however, the observation period was limited to 2 years [8]. In contrast, other prospective studies did not observe a significant benefit of the Kono-S anastomosis with regard to endoscopic recurrence compared with conventional anastomotic techniques [10, 11].

Furthermore, the multicenter retrospective analysis by Fichera et al., which included a median follow-up of 49 months, also failed to demonstrate a statistically significant difference between Kono-S and conventional anastomoses in terms of recurrence rates [12].

In our cohort, differences between groups in recurrence rates, postoperative complications, and operative time did not reach statistical significance. Nevertheless, the Kono-S group demonstrated slightly higher gastrointestinal quality-of-life scores (GIQLI = 116 vs. 110), without achieving statistical significance. The first randomized controlled trial of the Kono-S technique (the SuPREMe-CD study) likewise reported lower rates of endoscopic and clinical recurrence at 2 years; however, its limited follow-up duration and the use of a different quality-of-life instrument (IBDQ) restrict direct comparability [13].

Notably, Crohn’s disease–related rehospitalizations were numerically higher in the Kono-S group (5 vs. 1), although this difference did not reach statistical significance. Postoperative weight gain, another surrogate marker of clinical stability, was somewhat higher in the conventional group (6.3 kg vs. 2.3 kg), again without statistical significance. The overall incidence of postoperative complications, including anastomotic insufficiency and postoperative bleeding, was low and comparable between the two groups.

The clinical consequences of recurrence remain a central concern in postoperative management. In our series, the overall need for surgical reintervention was low: one patient in the Kono-S group and two patients in the conventional group required re-resection during long-term follow-up, with all reconstructions performed as side-to-side stapled anastomoses. This contrasts with historical series reporting cumulative re-resection rates of 30–40% after 10 years of follow-up. This discrepancy likely reflects a combination of aggressive postoperative medical therapy, early endoscopic management of strictures, and a substantial number of patients lost to follow-up (eight deaths and 37 patients not contactable), some of whom may have undergone re-resection elsewhere. Furthermore, rehospitalizations were infrequent; only one hospitalization was related to re-resection, whereas all other admissions were attributable to conservative or medical management of disease activity.

One aspect of the Kono-S anastomosis that warrants consideration is its structural configuration, which may facilitate endoscopic access in cases of anastomotic recurrence. Endoscopic balloon dilation is currently recommended as first-line therapy in such cases, and the functional end-to-end configuration of the Kono-S anastomosis may offer technical advantages. In our cohort, however, dilations were performed in both groups. Many dilations were conducted on an outpatient basis and were incompletely documented in the medical records, precluding reliable quantification. These limitations underscore the importance of standardized follow-up protocols and systematic documentation in future studies. From a practical perspective, the configuration of the anastomosis may play a subordinate role for the interventional endoscopist.

Furthermore, it remains debatable whether a technique associated with an additional learning curve should be widely adopted in the absence of a demonstrable advantage over established methods.

Importantly, approximately 40% of patients retrospectively indicated that they would have preferred surgery at an earlier stage of their disease, independent of the anastomotic technique employed. Although subject to recall bias, this observation highlights the importance of ongoing critical appraisal of surgical timing and interdisciplinary decision-making in the management of Crohn’s disease.

In summary, although the Kono-S technique did not result in statistically significant improvements in recurrence rates or postoperative outcomes, it remains a technically sound and safe alternative to conventional anastomotic procedures in ileocecal resection for Crohn’s disease. While it does not represent a breakthrough solution for recurrence prevention, it should be regarded as a valuable option within the surgical armamentarium for managing complex Crohn’s disease.

## Limitations

Several limitations should be acknowledged. First, this was a single-center observational study with a relatively small sample size, limiting statistical power and generalizability. Second, allocation of anastomotic technique was not randomized and depended on surgeon preference and intraoperative factors, introducing potential selection bias. Third, follow-up assessments were not standardized; colonoscopy and cross-sectional imaging were performed according to clinical indication rather than predefined intervals, which may have influenced detection of recurrence.

A proportion of the initial cohort could not be included in the final analysis due to death, or inability to establish contact, raising the possibility of attrition bias and underestimation of long-term recurrence and reoperation rates. Missing data were not imputed. In addition, baseline quality-of-life measurements were not available, precluding longitudinal assessment of functional improvement.

Finally, the exploratory nature of the study and the absence of formal sample size calculation limit the ability to detect small but potentially clinically relevant differences between techniques.

## Data Availability

The datasets generated and/or analyzed during the current study are not publicly available due to patient confidentiality and institutional restrictions, but are available from the corresponding author on reasonable request.
